# *Tsc1* Regulates the Proliferation Capacity of Bone-Marrow Derived Mesenchymal Stem Cells

**DOI:** 10.3390/cells9092072

**Published:** 2020-09-10

**Authors:** Maria V. Guijarro, Laura S. Danielson, Marta Cañamero, Akbar Nawab, Carolina Abrahan, Eva Hernando, Glyn D. Palmer

**Affiliations:** 1 Department of Pathology, NYU Grossman School of Medicine, NYU Langone Health, New York, NY 10016, USA; guijam@ufl.edu (M.V.G.); laurasdanielson@gmail.com (L.S.D.); 2 Department of Anatomy and Cell Biology, University of Florida, Gainesville, FL 32610, USA; akbarnawab@ufl.edu; 3 Roche Pharmaceutical Research and Early Development, Translational Medicine Oncology, Roche Innovation Center Penzberg, Nonnenwald, 282377 Penzberg, Germany; Marta.canamero@roche.com; 4 Department of Orthopaedics and Rehabilitation, University of Florida, Gainesville, FL 32610, USA; cabrahan@ufl.edu

**Keywords:** mesenchymal stem cell, TSC1, mammalian target of rapamycin (mTOR), senescence, stem cell proliferation, tuberous sclerosis

## Abstract

TSC1 is a tumor suppressor that inhibits cell growth via negative regulation of the mammalian target of rapamycin complex (mTORC1). *TSC1* mutations are associated with Tuberous Sclerosis Complex (TSC), characterized by multiple benign tumors of mesenchymal and epithelial origin. TSC1 modulates self-renewal and differentiation in hematopoietic stem cells; however, its effects on mesenchymal stem cells (MSCs) are unknown. We investigated the impact of *Tsc1* inactivation in murine bone marrow (BM)-MSCs, using tissue-specific, transgelin (*Tagln*)-mediated cre-recombination, targeting both BM-MSCs and smooth muscle cells. *Tsc1* mutants were viable, but homozygous inactivation led to a dwarfed appearance with TSC-like pathologies in multiple organs and reduced survival. In young (28 day old) mice, *Tsc1* deficiency-induced significant cell expansion of non-hematopoietic BM in vivo, and MSC colony-forming potential in vitro, that was normalized upon treatment with the mTOR inhibitor, everolimus. The hyperproliferative BM-MSC phenotype was lost in aged (1.5 yr) mice, and *Tsc1* inactivation was also accompanied by elevated ROS and increased senescence. ShRNA-mediated knockdown of *Tsc1* in BM-MSCs replicated the hyperproliferative BM-MSC phenotype and led to impaired adipogenic and myogenic differentiation. Our data show that *Tsc1* is a negative regulator of BM-MSC proliferation and support a pivotal role for the Tsc1-mTOR axis in the maintenance of the mesenchymal progenitor pool.

## 1. Introduction

Germline mutation of either *TSC1* (encoding hamartin) or *TSC2* (encoding tuberin) causes tuberous sclerosis (TSC), a multisystemic, autosomal dominant disorder with an estimated prevalence of 1 in 6000 newborns. TSC is characterized by benign, focal malformations called hamartomas, which comprise nonmalignant cells exhibiting abnormal cell proliferation and differentiation [1,2]. TSC often causes disabling neurological disorders, including epilepsy, mental retardation, and autism. Other major features of this syndrome include various manifestations of mesenchymal origin such as (1) renal angiomyolipomas [3], benign tumors composed of abnormal vessels, immature smooth muscle cells, and fat cells; (2) lymphangioleiomyomatosis, widespread pulmonary proliferation of abnormal smooth-muscle cells, and cystic changes within the lung parenchyma [4]; (3) cardiac rhabdomyomas, intracavitary or intramural tumors of striated cells that are present in nearly 50 to 70% of infants with TSC [5]. Loss of heterozygosity at the *TSC1* or *TSC2* locus and hyperphosphorylation of ribosomal protein S6 has been documented in each of the three cellular components of angiomyolipomas [6], suggesting that they may arise from a common progenitor and that the TSC1–TSC2 complex regulates the differentiation of cells that are derived from the mesenchyme.

TSC1 and TSC2 form a stable complex and function as the GTPase activating factor of the small GTPase Rheb. The Rheb cycles between a GTP-bound active form and a GDP-bound inactive form, and can potentially activate the mammalian target of Rapamycin complex 1 (mTORC1). Stimulation of Rheb GTP hydrolysis by the TSC1-TSC2 complex inhibits mTORC1 activity and downstream phosphorylation of its targets including, p70 S6 kinase (S6K) and eukaryotic translation-initiation factor 4E-binding protein 1 (4E-BP1), causing a reduction in cell growth and protein synthesis [7]. Persistent mTORC1 activation, resulting from genetic deletion of *TSC1* [8,9], *PTEN* [10], or overexpression of Wnt [11], has been shown to cause proliferative stem cell phenotypes in epithelial and hematopoietic tissues, followed by subsequent stem cell exhaustion. It has been proposed that aberrant mTORC1 activation drives stem cell depletion through the increased translation of downstream targets and subsequent activation of tumor-suppressive/fail-safe mechanisms resulting in cellular senescence or apoptosis [9,12,13,14]. However, the molecular mechanisms and targets of mTORC1 in this context are yet unknown. Interestingly, inhibition of mTORC1 also extends an organism’s lifespan [15,16], consistent with the notion that declining stem cell potential underlies aging [17].

Given the mesenchymal pathologies characteristic of TSC and the proposed roles of TSC1/TSC2 in stem cell maintenance, we investigated the effects of *Tsc1* inactivation in mesenchymal stem cells (MSCs, also defined as multipotent mesenchymal stromal/progenitor cells) and their derived progeny. MSCs comprise a heterogeneous subset of multipotent cells present in the stromal fraction of many adult tissues [18,19] that proliferate in vitro as plastic adherent fibroblast-like cells [20], exhibit colony-forming potential, and can differentiate into adipocytes, osteocytes, chondrocytes, fibroblasts, and myocytes [21]. However, despite their considerable therapeutic potential in a broad range of cellular therapies and tissue engineering protocols, cellular pathways that govern MSC self-renewal and maintenance in vivo remain poorly defined. Here, we describe the impact of *Tsc1* loss on the proliferative phenotype of bone marrow (BM)–MSCs in vitro and in vivo. Inactivation/suppression of *Tsc1* was achieved by either *Tagln*-Cre mediated knockout, which efficiently targets smooth muscle [22] and bone marrow progenitor populations [23], or shRNA-mediated knockdown. Organism lifespan, the proliferation of non-hematopoietic BM, MSC colony-forming potential, senescence, and cellular differentiation were evaluated in response to *Tsc1* loss, as well as mTOR contribution to those effects.

## 2. Materials and Methods

### 2.1. Generation of the Mouse Model

All animal experimentation and procedures were performed in accordance with NYU IACUC approved protocols (#061108-03 and #100108-01). Mice with smooth muscle-specific (Transgelin: *Tagln*) deletion of one or two alleles of *Tsc1* were generated by crossing mice with a conditional allele of *Tsc1^L/L^* (Tsc1tm1Djk/J, #005680) [24] with a *Tagln* allele expressing cre recombinase (Tg(Tagln-cre)1Her/J, [22] obtained from Jackson Laboratories (Bar Harbor, ME, USA). Heterozygous mice (*Tagln-cre^+^/Tsc1^Δ/+^*) were mated to generate homozygous *Tagln-cre^+^/Tsc1*^∆/∆^. Cohorts of *Tagln-cre^+^/Tsc1^+/+^*, *Tagln-cre^+^/Tsc1^Δ/+^,* and *Tagln-cre^+^/Tsc1*^∆/∆.^ were generated for bone marrow analysis in young (28 d) and old (1.5 yr) mice, and long-term survival studies (up to 2.3 yrs). To assess the distribution of *Tagln*-mediated recombination, *Tagln*-cre mice were bred with reporter mice containing the conditional RosaR26 β-galactosidase allele B6.129S4-Gt(Rosa)26Sortm1Sor/J (Rosa26-LSL-LacZ, 003 474 Jackson Labs) [25]. Tagln-Cre^+^/Rosa26-LacZ offspring were analyzed for recombination using β-galactosidase staining. Polymerase chain reaction (PCR) genotyping for conditional, null, and wild-type *Tsc1* alleles was performed on tail genomic DNA as previously described [26].

### 2.2. Isolation and Expansion of Mouse Mesenchymal Stem Cells (mMSCs)

Bone marrow (BM) was collected by flushing the long bones of murine tibias and femurs with MSC growth medium using an insulin syringe. BM was collected from pooled donors (*n* = 3) representing each genotype, and red blood cells were lysed by using ACK (Ammonium-Chloride-Potassium) Lysing Buffer. Cells were then centrifuged and washed with Hanks’ Balanced Salt solution (HBSS) prior to plating or analysis by flow cytometry. For expansion in monolayer, cells were plated at low density (100 cells/cm^2^) and cultured in complete isolation media (CIM), consisting of Roswell Park Memorial Institute 1640 Medium (RMPI 1640; Gibco, Carlsbad, CA, USA), 9% fetal bovine serum (FBS; Cell Gro, Manassas, VA, USA), 9% horse serum (HS; HyClone Thermo, South Logan, UT, USA), 100 U/mL penicillin/streptomycin (Thermo Scientific, Waltham, MA, USA) and 2 mM L-Glutamine (Invitrogen, Carlsbad, CA, USA). After 48 h, adherent cells were washed with PBS and fresh CIM was added every 3–4 days. After 2 weeks in culture, cells were detached with 0.25% trypsin (Gibco, Carlsbad, CA, USA) and plated in mMSC expansion media (CEM) consisting of Iscove’s Modified Dulbecco’s Medium (IMDM; Gibco, Carlsbad, CA, USA), 2 mM L-Glutamine, 10% FBS, 10% HS and 100 U/mL penicillin/streptomycin (Thermo Scientific, Waltham, MA, USA) for CFU-F assays, ROS measurements and β-gal staining.

For shRNA-mediated knockdown experiments, BM-derived MSCs were purchased from the Texas University Institute of Regenerative Medicine MSC Distribution Program (http://medicine.tamhsc.edu/irm/msc-distribution.html). The cells were referred to as wt BM-MSCs to distinguish from MSCs derived from control mice (*Tagln*-cre^+^/*Tsc1^+/+^*) used in transgenic knockout studies.

### 2.3. Flow Cytometric Analysis for Mmsc-Associated Cell Surface Markers

Primary BM isolates were depleted of Lin^-^ cells by negative immunomagnetic selection using the EasySep Biotin Selection kit (Stem Cell Technologies, Cambridge, MA, USA #18556) according to the manufacturer’s instructions. Biotin antibodies used for Lin positive selection were included in the Biotin-conjugated Mouse Lineage Panel (#559971 BD Pharmingen). Recovered cells were suspended in Dulbecco’s Modified Eagle Medium (DMEM) supplemented with 2% FBS and incubated with conjugated primary antibodies to MSC-associated cell surface markers for 30 min at room temperature. Unbound antibodies were removed by washing. Cells were detected using an LSRII FACSdiva flow cytometer (BD Biosciences). The antibodies used were CD54 (FITC-conjugated rat IgG2b), CD105 (PE-conjugated rat IgG2a, k), CD73 (PE-conjugated rat IgG1) (all from eBioscience, Thermofisher, Waltham, MA, USA), and CD106 (Alexa 488-conjugated rat IgG2a, k) (Biolegend, San Diego, CA, USA).

### 2.4. Bromodeoxyuridine (BrdU) Labeling

For assay of cell proliferation within the BM compartment in vivo, 1 mg of BrdU (BD Biosciences) was administered in a 100 µL volume (10 mg/mL) to control and *Tsc1* mutant mice via intraperitoneal injection 24 h prior to sacrifice and BM extraction. BrdU staining was performed according to the manufacturer’s instructions (FITC BrdU Flow Kit, BD Pharmingen #557891). Briefly, 10^6^ recovered Lin^-^MSC were fixed and permeabilized in BD Cytofix/Cytoperm buffer for 30 min on ice. After washing, cells were resuspended in BD Cytoperm buffer and incubated 10 min on ice. After further washing, cells were re-fixed in the BD buffer and finally treated with DNase to expose incorporated BrdU for 1 h at 37 °C. Cells were then stained with anti-BrdU antibody in BD Perm/Wash buffer for 20 min at room temperature. Cells positive for BrdU were detected using an LSRII FACSdiva flow cytometer (BD Biosciences).

### 2.5. Colony Forming Assay (CFU-F)

For CFU-F assays, 0.5 × 10^6^ primary BM cell isolates from each genotype were plated in 12 well plates in mMSC expansion medium for 9 days with media changes every 3rd day. To assess colony-forming potential after expansion, MSCs were replated at 10 and 50 cells/cm^2^. At the endpoint, cells were fixed with 1% glutaraldehyde (Thermo Scientific) and stained with 1% crystal violet (Sigma). The number of colonies per well was counted under a phase-contrast microscope. Triplicate measurements were performed for each genotype/treatment group. To assess the effects of the antioxidant N-Acetyl cysteine (NAC) on colony formation, primary BM cell isolates were cultured as above with the addition of 10 mM NAC (Sigma-Aldrich) to the mMSC expansion medium.

### 2.6. Flow Cytometry Analysis of Reactive Oxygen Species (ROS)

To measure the accumulation of ROS in control and *Tsc1* mutant MSCs, cells in monolayer culture were incubated with cell-permeable 2′,7′-dichlorodihydrofluorescein diacetate (DCF-DA, 10 µM) (Invitrogen) for 15 min at 37 °C following antibody staining for MSC-associated cell surface markers (Lin^-^, CD105^+^). Cells were trypsinized, washed, and analyzed by flow cytometry. MSCs with ROS accumulation were identified by gating in green (DCF-DA) and red (CD105) channels.

### 2.7. Mouse Histopathology and Immunohistochemistry

Mice were sacrificed at 28 days or 1.5 yrs for the harvesting of BM or tissue processing. Tissue samples were fixed in 10% neutral-buffered formalin, processed in graded alcohols, and embedded in paraffin according to standard protocols. Sections (5 µm) were prepared for hematoxylin and eosin (H&E) staining or antibody detection. Immunohistochemistry was conducted following the standard avidin-biotin immunoperoxidase staining procedure. Sections were blocked with the appropriate serum depending on the primary antibody (goat for rabbit IgG and horse for mouse IgG) and incubated with primary antibodies for p53 (CM5; Novocastra), pS6 (Cell Signaling Danvers, MA, USA, #9858), p16 (M-156; sc-1207), and p21 (C-19; sc-397G) (Santa Cruz). Diaminobenzidine (DAB) was used as the chromogen and hematoxylin was used to counterstain nuclei.

### 2.8. Senescence-Associated β-Galactosidase (SA β-G) Staining

#### 2.8.1. In Situ Whole Organ Staining

SAβG staining was performed on whole-mount kidneys isolated from control and *Tsc1* mutant mice following sacrifice at 28 days using the Senescence β-Galactosidase Staining Kit (Cell Signaling). Briefly, whole-mount kidneys were fixed at room temperature, for 2 h with a solution containing 2% formaldehyde and 0.2% glutaraldehyde in PBS, washed three times with PBS, and incubated overnight at 37 °C with the Staining Solution containing X-gal in N-N-dimethylformamide (pH 6.0). Kidneys were then dehydrated with two consecutive steps in 50% and 70% ethanol and embedded in paraffin for serial sectioning. Sections were counterstained with eosin.

#### 2.8.2. In Vitro Cell Culture

BM-MSCs derived from *Tsc1* mutant and control mice were seeded into 24-well plates (0.03 × 10^6^ cells/well) and cultured in MSC growth medium. After 36 days in culture, cells were washed with PBS, fixed with 4% formaldehyde in PBS for 15 min, and stained with the β-Galactosidase staining solution at pH 6.0, as described above. After overnight incubation at 37 °C, cells were analyzed for blue staining under a phase-contrast microscope.

### 2.9. Lentiviral shRNA Vector Generation and Transduction

Lentiviral shRNA-expressing constructs were generated for stable knockdown of *Tsc1* in wt BM-MSCs. GIPZ short hairpin lentiviral expression plasmids (Dharmacon, Lafayette CO) encoding sh*Tsc1* and non-silencing, scrambled (Scr) shRNA controls were co-transfected into 293T cells with the second-generation packaging plasmids pSPAX-2 (Addgene ID# 12260) and pMD2.G (Addgene ID# 12259). Viral supernatants were harvested 48 h after transfection and used directly for MSC transduction. A monolayer of MSCs was transduced 3× with viral supernatant on consecutive days in the presence of polybrene (8 µg/mL) prior to selection with 1 µg/mL puromycin for 2 weeks, to eliminate non-transduced cells.

### 2.10. Western Blot Analyses

Cell extracts from Scr- and sh*Tsc1*-transduced MSC cultures were lysed using RIPA buffer (Santa Cruz), supplemented with protease and phosphatase inhibitors (Roche, Basel, Switzerland). Protein quantification was performed using the Bradford Assay (Biorad) and 20 μg of sample lysate was resolved on 10% Novex Tris-Glycine SDS-PAGE gels (Invitrogen, Carlsbad, CA). Proteins were transferred to nitrocellulose membranes using iBlot (Invitrogen), blocked with 5% non-fat dry milk in Tris-buffered saline-Tween20 (0.1%) for 1 h, and incubated overnight at 4 °C with primary antibody, pS6(Ser235/236) (Cell Signaling, #4858) or GAPDH (Millipore, #ABS16). Membranes were washed and incubated with horseradish peroxidase-conjugated secondary antibodies for 30–60 min before development using Super Signal West Pico Plus Chemiluminescent substrate (Thermo Scientific) following the manufacturer’s recommendations. Membranes were scanned using Amersham Imager 680 for different times for optimal image analysis.

### 2.11. MSC Differentiation Assays

#### 2.11.1. Adipogenesis

ShRNA-transduced wt BM-MSCs were seeded at 70% confluence following puromycin selection, and incubated in an adipocyte induction medium [27], consisting of DMEM supplemented with 10% FBS, 0.25 µM dexamethasone, 10 µg/mL insulin, and 0.5 mM 3-Isobutyl-1-methylxanthine (IBMX) for 3 days. Media was then switched to maintenance medium, DMEM with 10% FBS and 10 µg/mL insulin, for additional 3 days. Adipogenesis was assessed by the RT-qPCR-based quantification of adipocyte markers and the appearance of lipid droplets detectable by Oil Red O staining after 6 days.

#### 2.11.2. Smooth Muscle (SM) Myogenesis

ShRNA-transduced MSCs were subjected to SM differentiation by incubation with DMEM containing 5% horse serum (HS) and 5 ng/mL recombinant human TGF-β1 (R&D Systems) for 6 days as previously reported by Uezumi et al. [27]. Differentiation was assessed by upregulation of smooth muscle markers quantified by RT-qPCR, and the formation of elongated, spindle-shaped cells, visualized by phase-contrast microscopy after 6 days.

### 2.12. RNA Extraction and RT-PCR

Total RNA was extracted from MSC cultured for 6 days in adipocyte or SM differentiation medium using Qiazol (Qiagen, Valencia, CA) followed by RNeasy Mini Kit (Qiagen, Valencia, CA, USA). cDNA was synthesized from 0.5 µg of total RNA using the Taqman Assay kit (Applied Biosystems, Austin, TX, USA) and amplified by real-time PCR using gene-specific primers designed from murine gene sequences (Table 1) on a BioRad iCycler (BioRad, Hercules, CA, USA) using FastStart SYBR Green MasterMix (Roche).

### 2.13. Everolimus Treatment

Everolimus (RAD001; LC Labs) was administered via intraperitoneal injection to pregnant females of *Tsc1^+/+^*, *Tsc1^∆/+^*, and *Tsc1^∆/∆^* genotypes during the last 12 days of pregnancy (0.2 µg/g/day). Treatment was continued daily (2 µg/g/day) in newborns up to 28 days until sacrifice. For in vitro MSC studies, everolimus was added to MSC growth medium (20 nM, final concentration), with media changes every 3 days.

### 2.14. Statistical Analyses

Statistical significance was determined using GraphPad Prism Software. Overall survival was analyzed by Kaplan–Meier curves and the log-rank (Mantel–Cox) test was used to determine differences in survival. Statistical significance was defined as *p* < 0.05. One-way Anova was used to compare more than 2 groups; 2-tailed Student’s t-tests were used to compare time points.

## 3. Results

### 3.1. Tagln-Mediated Tsc1 Inactivation Targets SM and MSC Populations and Recapitulates Features of Human Tuberous Sclerosis

**Figure 1:**
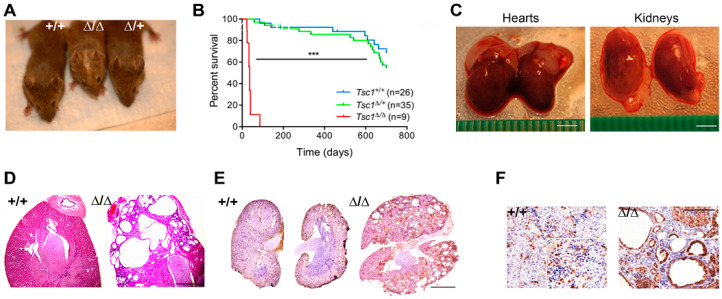
*Tagln*-mediated *Tsc1* inactivation replicate features of human tuberous sclerosis. (**A**) Representative macroscopic pictures of *Tsc1^+/+^*, *Tsc1^∆/+^,* and *Tsc1^∆/∆^* 28 day old littermates. Note the reduced size of *Tsc1^∆/∆^* mouse (middle) and white hair on the head. (**B**) Survival curve for mice with the indicated genotypes as a function of days. A statistically significant decrease in lifespan was observed for *Tsc1^∆/∆^* mice compared with *Tsc1^∆/+^* and *Tsc1^+/+^* cohorts (***, *p* < 0.001). (**C**) Hearts and kidneys from *Tsc1^+/+^* control and *Tsc1^∆/∆^* knockout mice indicating a modest enlargement of both organs following *Tsc1* inactivation. (**D**) Hematoxylin and eosin (H&E) staining of sections of kidneys isolated from 28 day old *Tsc1^∆/+^* and *Tsc1^+/+^* mice, revealing a spongiotic renal appearance and bilateral cystification following homozygous *Tsc1* deletion (∆/∆). Scale bar: 1000 μm. (**E**) pS6 staining (brown color) of *Tsc1^+/+^* and *Tsc1^∆/∆^* kidneys indicating increased activation of mTOR following *Tsc1* inactivation. Scale bar: 2000 μm. (**F**) Higher magnification views showing localization of pS6 staining within lining cells of cystic lesions in *Tsc1^∆/∆^* kidneys. Scale Bar: 250 μm.

To investigate the in vivo effects of *Tsc1* inactivation, we used a *Tagln*-driven conditional knockout approach to enable tissue-specific targeting of smooth muscle and mesenchymal progenitors and overcome early embryonic lethality associated with global *Tsc1* deletion. We, therefore, crossbred *Tsc1*^L/L^ [24] and *Tagln-cre* [28] mice to obtain heterozygous *Tagln*-cre^+/-^*Tsc1*^Δ/+^ siblings to generate mice with deletion of none, one or two alleles of *Tsc1.* The homozygous inactivation of *Tsc1* (hereafter *Tsc1^∆/∆^*) in the SM lineage was able to bypass the embryonic lethality characteristic of *Tsc1* knockout mice [29]. However, homozygous mice had a dwarfed appearance with signs of premature aging at 28 d (white-haired in the upper head and hair loss in certain body areas) (Figure 1A), and dramatically shortened lifespan (average = 30 ± 6.8 days) compared to that of control mice (hereafter, *Tsc1^+/+^*) (624 ± 32 days; *p* < 0.001) or heterozygous mice (hereafter *Tsc1^∆/+^*) (606 ± 30 days; *p* < 0.001) (Figure 1B). *Tsc1^∆/∆^* mice presented macroscopically bigger hearts and kidneys compared to *Tsc1^+/+^* mice (Figure 1C). These findings resemble clinical manifestations of human TSC and agree with the phenotypes reported by Malhowski et al. following *SM22α–*(*Tagln*) mediated deletion of *Tsc1* [30].

Kidneys from *Tsc1^∆/∆^* mice also exhibited multiple cystadenomas (Figure 1D,E) and increased immunostaining of phosphorylated ribosomal protein S6 (pS6) compared to *Tsc1^+/+^* mice (Figure 1E,F), consistent with constitutive mTOR activation following *Tsc1* deletion. Tissue specificity of *Tagln*-mediated recombination within the smooth muscle lineage was confirmed by crossing *Tagln-cre* mice with *Rosa26-LSL-*LacZ reporter mice [25] and assessing recovered tissues for β-gal staining. LacZ signal was observed in the kidneys (Supplementary Figure S1) as well as other smooth muscle tissues as previously described [31], but absent in other cell types. The close correspondence between lacZ staining in the kidneys of *Tagln*-*cre*-lacZ reporter mice (Supplementary Figure S1) and pS6 staining in *Tsc1^∆/∆^* mice (Figure 1F) suggest that mTORC1 activation arises from cells harboring *Tsc1* deletion.

In addition to SM, we previously demonstrated targeted recombination within non-hematopoietic BM by crossing *Tagln*-cre mice with *Rosa26*-EGFP mice [32]. Flow cytometry of BM mononuclear cells revealed GFP expression in a subpopulation (6.46% ± 0.52%) of Lin^-^c-kit^-^Sca1^+^ cells, which encompass the MSC pool [33], indicating *Tagln*-mediated recombination [31]. Based on these observations, the effects of *Tsc1* inactivation on the BM-MSC phenotype were further explored in vitro and in vivo.

### 3.2. Tsc1 Deletion Leads to Expansion of the BM-MSC Pool in Young (28 Day Old) Mice

**Figure 2:**
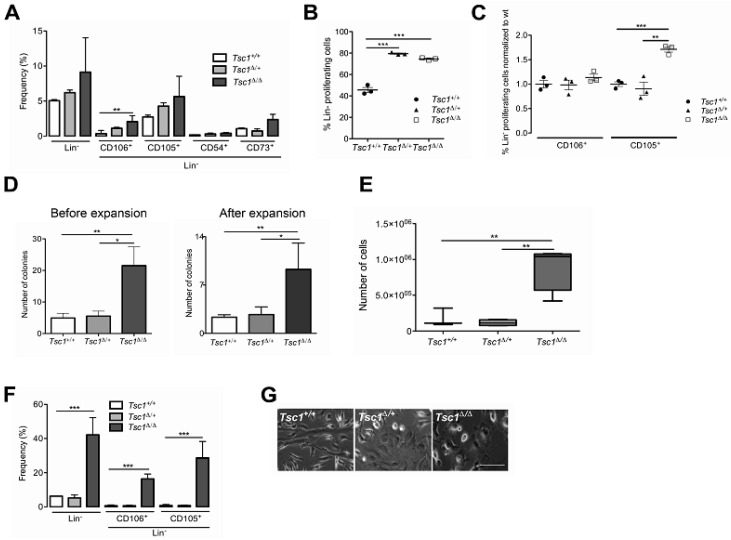
*Tsc1* inactivation targets bone marrow mesenchymal stem cells (BM-MSCs) and leads to enhanced proliferation in vivo and in vitro. (**A**) Cell population frequency, determined by flow cytometry of *Tsc1^+/+^*, *Tsc1*^Δ/+^, and *Tsc1*^Δ/Δ^ BM-Lin^-^ cells immediately following isolation from 28 day old mice, showing Lin^-^ and Lin^-^ CD106, -CD105, -CD54, and -CD73 subpopulations. (**B**) Frequency of proliferating BM-Lin^-^ cells, determined by BrdU incorporation, in control and *Tsc1* mutant mice. (**C**) Percentage of proliferative cells within CD106^+^ and CD105^+^ subpopulations of BM-Lin^-^ cells. (**D**) CFU-F assay determining colony forming potentials of BM-MSCs from each genotype. Assays were performed by directly plating primary BM Lin^-^ cell isolates (Before expansion) or replating expanded MSCs after 20 days in culture (After expansion). (**E**) The number of adherent cells following in vitro expansion after 20 days. (**F**) Frequency of Lin^-^, Lin^-^CD105^+,^ and Lin^-^CD106^+^ cells after 20 days of in vitro expansion.) (**G**) Representative bright-field images of adherent cells from each genotype after 13 days in culture. Panels A–C: Data shown are means ± SD, *n* = 3 mice per group. Scale bar: 100 µm. Panels D–F: Data are triplicate measurements of pooled BM-MSCs from each genotype *, *p* < 0.05; **, *p* < 0.01; ***, *p* < 0.001.

Given previous reports of mTOR-dependent regulation of stem cell renewal and proliferation [8,9,10,11], we investigated the impact of *Tsc1* inactivation within the non-hematopoietic BM compartment. *Tagln*-mediated deletion of *Tsc1* in BM-MSCs was confirmed by PCR of genomic DNA from culture-expanded MSCs derived from Lin- BM isolated from young (28 day) *Tsc1^∆/∆^*, *Tsc1^∆/+^* and *Tsc1^+/+^* mice using primers specific for *Tsc1* floxed loci. An amplified product indicating *Tsc1* deletion was obtained in BM-Lin^-^ cells from *Tsc1^∆/∆^* and *Tsc1^∆/+^* mice, but absent from *Tsc1^+/+^* controls (Supplemental Figure S2A). To determine the effects of *Tsc1* inactivation on the proliferation of the BM-MSC pool in vivo, we isolated BM-derived mononuclear cells from each genotype in 28 day old mice and performed flow cytometry for cell surface markers associated with MSCs. A total of four markers were selected (CD105, CD106, CD54, CD73) which are commonly expressed in plastic-adherent culture [34,35,36] or used to classify an MSC phenotype [37]. A greater percentage of Lin^-^, CD106^+^, CD105^+^, CD54^+^, and CD73^+^ populations was found in *Tsc1^∆/∆^* mice compared to *Tsc1^∆/+^* and *Tsc1^+/+^* mice (Figure 2A). Moreover, except for CD73, the levels of MSC markers in heterozygous, *Tsc1^∆/+^* MSCs were intermediate between *Tsc1^∆/∆^* and *Tsc1^+/+^* mice, indicating gene dose-dependent effects (Figure 2A).

To determine whether the increased cellularity within the BM compartment following *Tsc1* inactivation was due to enhanced proliferation in vivo, we injected mice with 5-bromo-2′-deoxyuridine (BrdU) and analyzed cell surface marker expression after 24 h. Flow cytometry of recovered BM revealed a higher rate of proliferation of Lin^-^ cells from *Tsc1^∆/∆^* (*p* < 0.05) and *Tsc1^∆/+^* (*p* < 0.05) mice compared to *Tsc1^+/+^* (Figure 2B). Among Lin^-^ cells, there was a significantly higher rate of BrdU incorporation within the CD105^+^ subpopulation compared to *Tsc1^+/+^* (*p* = 0.0007) and heterozygous littermates (*p* = 0.0051) (Figure 2C). In contrast, *Tsc1* inactivation did not affect the proliferation of the CD106^+^ subpopulation of Lin^-^ BM in vivo (Figure 2C).

As the MSC phenotype is primarily characterized by a fibroblast-like appearance on tissue culture plastic and colony-forming capacity, we next investigated the effects of *Tsc1* inactivation on adherent cell growth in vitro. Primary BM isolates obtained from pooled donors of each genotype were seeded onto 6-well plates and cultured in mMSC expansion medium. After 9 days in culture, cells had acquired a typical spindle-shaped morphology characteristic of MSCs, and homozygous deletion of *Tsc1* led to a ~4-fold increase in the number of colonies compared to heterozygous (*p* = 0.0132) and *Tsc1^+/+^* cells (*p* = 0.009) (Figure 2D, left graph). To determine whether MSCs retained colony-forming capacity following expansion, cells were cultured for a further 20 days, and CFU-F assays were repeated on expanded cells. Following expansion, colony-forming capacity in *Tsc1^∆/∆^* remained significantly (~3-fold) higher than control (*p* = 0.009) and *Tsc1^∆/+^* groups (*p* < 0.05) (Figure 2D, right graph) and cell counts revealed a ~10-fold increase in *Tsc1^∆/∆^*-derived cells compared to the other genotypes (*p* < 0.05) (Figure 2E)**,** including a higher percentage of Lin^-^CD105^+^ (*p* < 0.0001) and Lin^-^CD106^+^ (*P* = 0.0002) populations (Figure 2F). Phase-contrast microscopy revealed morphological differences across cells of different genotypes, with *Tsc1* deficient cells exhibiting a more rounded polygonal shape compared to *Tsc1^+/+^* cells (Figure 2G). Together these data indicate that within young mice, *Tagln*-Cre mediated *Tsc1* inactivation induces expansion of non-hematopoietic BM in vivo and increased BM-MSC colony number and proliferation in vitro.

### 3.3. Aged (1.5 yr old) Mice Do Not Exhibit A Hyperproliferative BM-MSC Phenotype Following Tsc1 Loss

**Figure 3:**
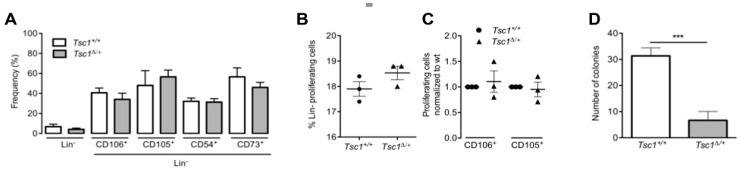
The population of BM-MSCs is no longer expanded in *Tsc1*^Δ/+^ aged mice. (**A**) Cell population frequency, by flow cytometry of BM-Lin^-^ cells immediately after isolation from 1.5 yr old Tsc1^+/+^ and Tsc1^Δ/+^ mice. Lin^-^ and Lin^-^CD106^+^, -CD105^+^, -CD54^+^, and -CD73^+^ subpopulations are shown. (**B**) Percentage of proliferating BM-Lin^-^ cells, by BrdU incorporation, in Tsc1^+/+^ and Tsc1^Δ/+^1.5 yr old mice. (**C**) Percentage of proliferating cells within Lin^-^CD106^+^ and Lin^-^CD105^+^ subpopulations. (**D**) CFU-F assay of BM-Lin^-^ cells isolated from 1.5 yr old mice. Panels A–C: Data shown are means ± SD, *n* = 3 mice per group. Panel D: Data are triplicate measurements of pooled BM-MSCs for both genotypes ***, *p* < 0.001.

Prolonged activation of intracellular signaling pathways including PI3K/AKT and mTOR has been found to be associated with loss of stem cell quiescence and depletion of the stem cell pool in vivo [9,11,38]. We, therefore, investigated whether sustained inactivation of *Tsc1*—a negative regulator of mTOR—impacts BM-MSC by assessing proliferation in aged (1.5 yr old) mice. As total loss of *Tsc1* (*Tsc1^∆/∆^)* induced lethality 30 ± 6.8 days after birth, only BM-MSC isolated from heterozygous, *Tsc1^∆/+^*, and control *Tsc1^+/+^* were available for study. Using separate cohorts from the studies in Figure 1, *Tsc1* recombination in aged *Tsc1^∆/+^* mice was confirmed by RT-PCR of cultured MSCs derived from Lin^-^ BM (Supplemental Figure S2B). Flow cytometry revealed that the frequency of BM-Lin^-^ cells in 1.5 yr old mice was roughly equivalent between *Tsc1^∆/+^*, and *Tsc1^+/+^* mice (Figure 3A). This contrasted with 28 day old mice (Figure 2A), in which modest increases were observed for Lin^-^, CD105^+^, and CD106^+^ populations in heterozygous *Tsc1^∆/+^* mice compared to *Tsc1^+/+^* controls. Similarly, Lin^-^ proliferation, measured by BrdU incorporation, was not significantly different in *Tsc1^∆/+^* and *Tsc1^+/+^* aged mice (Figure 3B), compared with a ~2-fold increase in *Tsc1^∆/+^* at 28 days (*p* < 0.05) (Figure 2B) indicating loss of hyperproliferation of non-hematopoietic BM cells with aging. Within Lin/CD106^+^ and Lin^-^/CD105^+^ subpopulations, loss of one *Tsc1* allele did not affect proliferation, as BrdU incorporation levels were similar among both genotypes, regardless of age (Figure 3C and Figure 2C). Importantly, BM-MSC from *Tsc1^∆/+^* aged mice exhibited reduced colony-forming potential in vitro: CFU-F assay revealed a ~5-fold decrease in colony number in *Tsc1^∆/+^* mice relative to *Tsc1^+/+^* (*p* < 0.0001) (Figure 3D) while at 28 days, colony number was roughly equivalent between the 2 genotypes (Figure 2D). These findings provide evidence that non-hematopoietic BM hyperproliferation, associated with *Tsc1* loss is age-dependent, with prolonged suppression of *Tsc1* (*Tsc1^∆/+^*) resulting in fewer colony-forming BM-MSCs. This may reflect the depletion of the BM stem cell pool following unrestricted proliferative expansion at earlier developmental stages.

### 3.4. Tsc1 Inactivation Leads to ROS Production and Senescence

**Figure 4:**
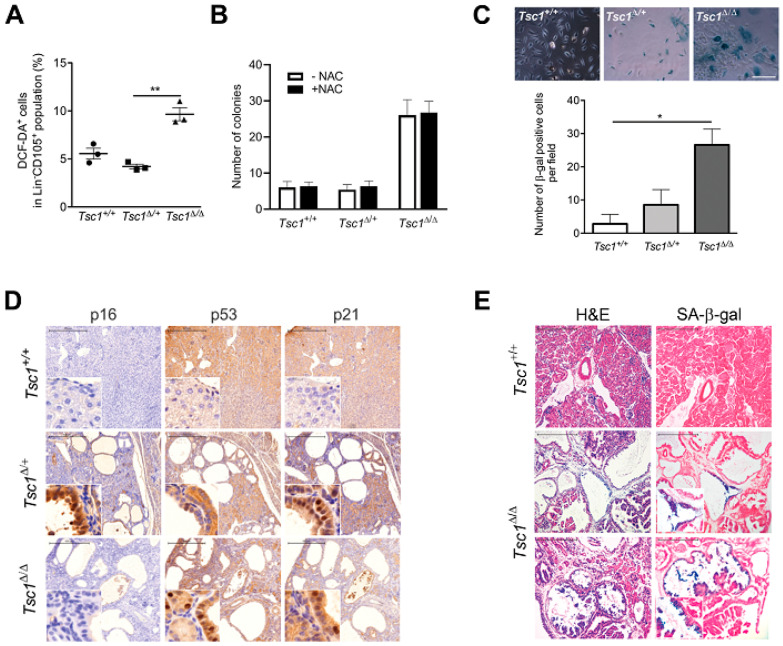
*Tsc1* suppression leads to ROS production and premature senescence. (**A**) Levels of ROS in monolayer cultures of Lin^-^CD105^+^ cells derived from *Tsc1^+/+^*, *Tsc1*^Δ/+^, and *Tsc1*^Δ/Δ^ 28 d old mice determined by flow cytometry following incubation with DCF-DA. Data presented are means ± SD (*n* = 3 animals per group). (**B**) CFU-F assay of primary BM Lin^-^ isolates in the presence or absence of the antioxidant NAC. (**C**) SA β-gal staining following extended culture (36 days) of BM-MSCs. Images show SA β-gal positive cells for each genotype. Scale bar: 100 μm. The plot indicates the quantification of SA β-gal positive cells. Values represent average counts of 5 fields for each genotype. (**D**) Representative images of sections of *Tsc1^+/+^*, *Tsc1*^Δ/+^, and *Tsc1*^Δ/Δ^ kidneys from 28 day old littermates stained for p16, p21, and p53. Scale bar: 500 µm. (**E**) Representative images of *Tsc1^+/+^* and *Tsc1*^Δ/Δ^ kidneys stained with H&E or SA β-gal. Scale bar: 250 µm. Inset images show 10× (panel D) and 4× (panel E) magnifications of hyperproliferative cystic regions with positive staining for cell senescent markers.* *p* < 0.05; ** *p* < 0.01.

In addition to stem cell exhaustion, persistent elevation of mTOR activity associated with *Tsc1* deficiency has also been linked to the production of reactive oxygen species (ROS) in hematopoietic stem cells (HSCs), leading to hyperproliferation, followed by a rapid decline in stem cell self-renewal [8]. To determine if *Tsc1* inactivation affects ROS production in MSCs, BM-MSCs isolated from young 28 day old *Tsc1^+/+^*, *Tsc1^∆/+^*, and *Tsc1^∆/∆^* mice were analyzed by flow cytometry upon incubation with 2′-7′-Dichlorofluorescin diacetate (DCF-DA), a substrate that becomes fluorescent when reacting with ROS. As shown in Figure 4A, levels of DCF-DA were 1.8-fold higher in Lin^-^CD105^+^ cells from *Tsc1^∆/∆^* mice compared to *Tsc1^∆/+^* and *Tsc1^+/+^* (*p* = 0.0129), consistent with their higher proliferative activity (Figure 2C). To determine if the increased clonogenicity shown by *Tsc1*-deficient MSCs was attributable to ROS production, MSCs from each genotype were treated with the antioxidant N-acetyl-cysteine (NAC). NAC treatment did not affect colony formation for any of the genotypes, and colony number remained higher in *Tsc1^∆/∆^* indicating that increased ROS levels in *Tsc1^∆/∆^* do not account for their higher clonogenic capacity (Figure 4B).

To investigate whether the enhanced proliferation of *Tsc1^∆/∆^* MSCs leads to premature senescence [13,14], cells of each genotype were expanded in monolayer culture for 6 weeks and stained for senescence-associated β-galactosidase (SA β-gal). After extended culture, the number of β-gal positive cells, which exhibited a mostly large-flat morphology, increased with the extent of *Tsc1* inactivation (Figure 4C). In *Tsc1^∆/∆^* MSCs, there was a ~15-fold increase in β-gal positive cells relative to the *Tsc1^+/+^* controls (*p* = 0.0161) (Figure 4C).

To determine whether *Tsc1* inactivation induces a senescent phenotype in vivo, we performed immunostaining for cell senescent markers (p16, p53, and p21) in the kidneys, which were found to display significant hyperproliferation and cell dysplasia following *Tsc1* deletion. In young mice (28 day old) abundant staining for p53, and its downstream target p21, was observed in the kidneys of both *Tsc1^∆/+^* and *Tsc1^∆/∆^* genotypes, and absent from *Tsc1^+/+^* controls (Figure 4D). Interestingly, staining for p16 was observed only in *Tsc1^∆/+^* mice suggesting that *Tsc1* downregulation, but not a complete knockout, is associated with p16-mediated senescence. SA β-gal staining of *Tsc1^∆/∆^* kidneys also identified senescent cells lining cystic lesions that co-localized with areas of hyperproliferation (Figure 4E, lower panels), and were absent in *Tsc1^+/+^* controls (Figure 4E, upper panels). Together, these findings indicate that the proliferative phenotype associated with *Tsc1* inactivation is also accompanied by an onset of cellular senescence in both BM-MSCs and SM compartments consistent with premature cell aging.

### 3.5. Tsc1 Knockdown in Wt BM-Mscs Increases Their Clonogenic Potential and Suppresses Adipocyte and Smooth Muscle Differentiation In Vitro

**Figure 5:**
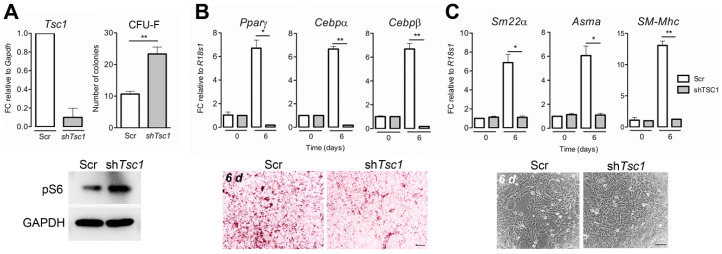
shRNA-mediated knockdown of *Tsc1* in wt BM-MSCs enhances clonogenicity and suppresses adipocyte and SM differentiation. (**A**) Relative *Tsc1* mRNA levels, colony number (CFU-F) and pS6 protein levels in mouse (C57BL/6) BM-MSCs were transduced with sh*Tsc1* and Scr sequences. *Gapdh* was used as a housekeeping control for RT-PCR and immunoblot. (**B**) Adipocyte differentiation of sh*Tsc1-* and Scr-transduced BM-MSCs. Relative mRNA levels of *Pparγ*, *Cebpα,* and *Cebpβ* before (day 0) and after 6 days of adipogenic stimulation (day 6). *r18s1* was used as a housekeeping control. Images (below) show oil red o staining of transduced BM-MSCs after 6 days of adipogenic differentiation. Scale bar: 100 µm. (**C**) Smooth muscle differentiation of *shTsc1-* and Scr-transduced BM-MSCs. Relative mRNA levels of *Sm22α*, *Asma*, and *SM-Mhc* before and after 6 days of myogenic stimulation. The images (below) show the cellular organization of transduced BM-MSCs after 6 days of SM differentiation. Scale bar: 100 µm. Data presented are means ± SD, *n* = 3, per group. * *p* < 0.05; ** *p* < 0.01.

To investigate whether the hyperproliferative phenotype associated with BM-MSCs in *Tsc1* deficient mice can be conferred to non-transgenic, post-natal, wt MSCs, we performed shRNA-mediated *Tsc1* knockdown. BM-MSCs obtained from C57BL/6 mice were transduced with shRNA-lentiviral vectors expressing either sh*Tsc1* or a non-silencing, scrambled sequence (Scr) and assessed for proliferation and differentiation in vitro. The expression of sh*Tsc1* led to a ~90% knockdown of *Tsc1* mRNA levels compared to controls (Figure 5A). In a CFU-F assay, *Tsc1* knockdown resulted in a greater than 2-fold increase in the number of colonies compared to control cells, mirroring the effects of *Tagln*-Cre mediated *Tsc1* inactivation (*p* = 0.0058) (Figure 5A). Immunoblot of cell extracts from Scr- and sh*Tsc1*-transduced MSCs also revealed elevated S6 phosphorylation (Ser 235/236) (Figure 5A), consistent with increased pS6 staining observed in tissues harvested from *Tsc1*^∆/∆^ mice (Figure 1E,F).

To address the potential effects of *Tsc1* knockdown on MSC differentiation capacity, sh*Tsc1*-transduced MSCs were induced to differentiate into adipocytic and smooth muscle lineages (Figure 5C,D). *Tsc1* knockdown blocked the upregulation of transcription factors, *Pparγ* (*p* = 0.018), *Cebpα* (*p* = 0.0011), or *Cebpβ* (*p* = 0.0049), observed in control cells after 6 days in adipocytic differentiation media, as shown by semi-quantitative RT-PCR (Figure 5C). Accordingly, intracellular lipid vacuole formation, characteristic of mature adipocytes, was evident in both cultures following adipogenic stimulation, however, controls exhibited more extensive Oil Red O staining, compared to *Tsc1* knockdown cells (Figure 5C).

Likewise, when exposed to smooth muscle differentiation media, sh*Tsc1*-transduced cells did not show the upregulation of early markers of SM differentiation, *Sm22α* (*p* = 0.022) and *Asma* (*p* = 0.0252) seen in controls, nor *SM*-*Mhc*, an unequivocal marker of mature SM cells (*p* = 0.034) (Figure 5C). Differences in cell morphology were also evident following SM differentiation, with more elongated cells apparent in Scr-transduced controls, while *Tsc1* knockdown cultures contained proliferative compact cells (Figure 5C).

### 3.6. mTOR Activation is Required for BM-MSC Expansion Following Tsc1 Inactivation

**Figure 6:**
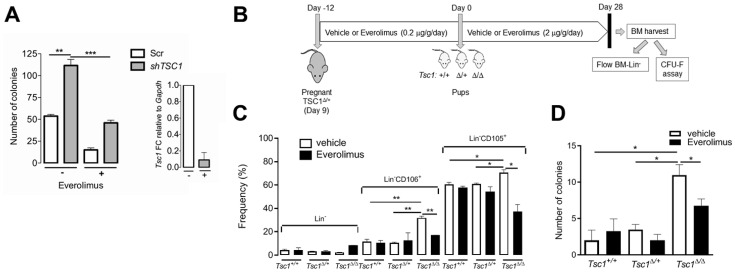
Everolimus inhibits the proliferative expansion of BM-Lin^-^ cells and MSCs following *Tsc1* inactivation. (**A**) CFU-F assay showing colony number in sh*Tsc1-* or Scr-transduced wt BM-MSCs with (+) and without (-) everolimus treatment (20 nM). Insert graph shows corresponding *Tsc1* mRNA levels (fold change) in shRNA transduced cultures. (**B**) Experimental scheme for investigation of BM hyperproliferation in *Tsc1* mutants following in vivo treatment with everolimus. (**C**) Cell population frequency, by flow cytometry of BM-Lin^-^ cells immediately the following isolation from 28 day old pups with and without everolimus treatment. Lin^-^ and Lin^-^CD106^+^ and -CD105^+^ populations are shown for each genotype. (**D**) CFU-F assay showing colony number of BM-Lin^-^ MSCs of each genotype following in vivo administration of everolimus or vehicle controls. Panel A: Data show means ± SD, *n* = 3 per group. Panels C–D: Data show means ± SD, *n* = 3 animals per group. *, *p* < 0.05; **, *p* < 0.01; ***, *p* < 0.001.

*Tsc1* is a known negative regulator of mTOR activity. Therefore, we investigated mTORcontribution to the hyperproliferative phenotype of BM-MSCs following *Tsc1* suppression/inactivation. In CFU-F assays, colony-forming potential was compared in wt BM-MSCs transduced with control (Scr) or *Tsc1* shRNAs in the presence of the mTOR inhibitor, everolimus (Figure 6A). While *Tsc1* knockdown increased colony number >2-fold (*p* = 0.013) consistent with the data shown in Figure 5A, concomitant drug treatment reduced the number of colonies to Scr levels (Figure 6A) (*p* = 0.0003). Colony number was also reduced by everolimus treatment within Scr controls, likely reflecting baseline activation of mTOR in MSCs under standard culture conditions.

To determine whether mTOR inactivation can also reverse the expansion of the BM stem cell compartment characteristic of in vivo *Tsc1* knockout, we injected everolimus (0.2 µg/g/day) into pregnant *Tsc1^∆/+^* females of each genotype during the last 12 days of pregnancy, and the perinatal offspring (2 µg/g/day). At 28 days after birth, mice of each genotype were sacrificed and BM was harvested for analysis (Figure 6B). *Tsc1^∆/∆^* mice had a significantly increased percentage of cells positive for CD106 and CD105 compared to *Tsc1^+/+^* and *Tsc1^∆/+^* mice in the vehicle treatment group (Figure 6C), consistent with the findings reported in Figure 2A. Following everolimus treatment, the percentages of both CD105 and CD106 populations in *Tsc1^∆/∆^* mice were significantly reduced compared to vehicle controls (*p* = 0.0039 and *p* = 0.0301, respectively) reaching levels comparable to that of non-treated, *Tsc1^+/+^* mice (Figure 6C). Similarly, colony-forming potential of BM-isolates was significantly increased in *Tsc1^∆/∆^* mice relative to the other genotypes in vehicle-treated controls (Figure 6D), consistent with the findings in Figure 2D, but everolimus treatment led to a significant (*p* = 0.0108) reduction in colony number (Figure 6D).

Taken together, our findings indicate that everolimus can abrogate the effects of *Tsc1* inactivation on the expansion of CD105^+^ and CD106^+^ cells in vivo, and MSC clonogenic potential in vitro. Thus, activation of the mTOR pathway is, at least in part, required for the hyperproliferative phenotype of non-hematopoietic BM cells and colony-forming MSCs following down-regulation or inactivation of *Tsc1*.

## 4. Discussion

*Tsc1* is an essential and specific negative regulator of mTOR signaling, which controls cellular growth, differentiation, and energy response [39]. Our studies show, through gene deletion and shRNA knockdown studies, that *Tsc1* controls proliferation, self-renewal, and differentiation capacity of the MSC pool in the BM. For gene inactivation, we employed *Tagln*-mediated recombination which was found to target both smooth muscle containing organs as well as non-hematopoietic bone marrow, confirming our previous observations [31]. The resulting phenotype exhibited many of the clinical manifestations of TSC, including cystadenomatous tumors in the kidneys and cardiac hypertrophy. These findings also replicated pathologies previously described for *Tagln*-mediated inactivation of *Tsc1* [30], as well as other genetic models adopting global or conditional knockout approaches [24,40]. Homozygous *Tsc1* inactivation also caused a dramatic increase in mortality, suggesting possible acute organ failure. While the life expectancy for clinical TSC is not known, renal disease and brain tumors have been identified as major causes of mortality in patient studies [41,42]. In *Tsc1*-deficient mice, premature death has been linked to cardiac dysfunction, however, this has not been definitively determined [30]. We also found that systemic administration of everolimus, a selective inhibitor of the mTORC1 complex, reversed the proliferative BM phenotype and increased clonogenicity of MSCs mediated by *Tsc1* loss, supporting a pivotal role for constitutive mTOR activation in maintenance stem cell proliferation in vivo.

Our data show, for the first time, a BM-MSC phenotype following *Tagln* mediated *Tsc1* inactivation. The absence of *Tsc1* was found to induce the proliferative expansion of non-hematopoietic BM cells (Lin^-^, CD105^+^ CD106^+^, and CD73^+^ cell populations) and increase the colony-forming potential of adherent bone marrow-derived cells, suggesting enhanced MSC self-renewal capacity. While no specific cell surface marker or combinations can distinguish multipotent MSCs either in vitro or in vivo, the absence of hematopoietic markers (Lin^-^), and expression of CD105 (endoglin) and CD73 (Ecto-5-Nucleotidase) are part of the recommended minimum criteria for the definition of human MSCs by the International Society of Cell Therapy [37]. In C57BL/6J mice, enhanced BM-MSC proliferation and multipotency have been found to associate with a CD105^+^ subpopulation [43] and CD73 expression [44]. Additionally, CD106 (Vascular cell adhesion molecule-1) has been identified as a cell-surface marker of BM-MSCs in humans [45] and mice, although expression is variable among inbred strains [34]. On the other hand, classic colony-forming (CFU-F) assays, initially described by Friedenstein et al. [46,47] remain a widely used indicator of MSC progenitor numbers, and CFU-F frequency is strongly linked to MSC differentiation capacity [48].

We also show that the hyperproliferative phenotype of *Tsc1*-deficient MSCs can be reproduced in wt cells by shRNA-mediated *Tsc1* knockdown. However, despite their enhanced colony-forming capacity, sh*Tsc1*-BM-MSCs displayed reduced adipogenesis and smooth muscle myogenesis in vitro, indicating impaired cellular differentiation. These observations are consistent with *Tsc1*/*Tsc2* inactivation in mouse embryonic fibroblasts (MEFs) causing hyperactivation of mTOR, and inhibition of myogenic and adipogenic differentiation via STAT3/p63/Notch signaling [49]. Conversely, mTOR inhibition using rapamycin has been shown to enhance smooth muscle cell differentiation and inhibit MSC proliferation via differential regulation of PI3K effectors, Akt2 and S6K [50,51]. Recent studies of targeted *Tsc1* deletion have also revealed impaired osteo- and chondro-lineage differentiation resulting from mTOR hyperactivation. Ablation of *Tsc1* in mesenchymal progenitors by *Prx1-Cre* revealed increased proliferation of MSCs and impaired osteoblast differentiation culminating in reduced mineralization and bone quality [52]. Similarly, in developing growth plates, *Col2*-mediated *Tsc1* ablation disrupted endochondral bone growth through increased chondrocyte proliferation and impaired hypertrophy and terminal maturation [53]. These reports, in combination with our own findings, suggest that mTOR hyperactivation, resulting from *Tsc1* loss, uncouples the normal proliferation and differentiation programs in mesenchymal progenitors, leading to impaired terminal maturation and defective development of musculoskeletal and smooth muscle tissues.

Analysis of *Tsc1^∆/+^* in aged mice revealed the loss of the hyperproliferative phenotype within the BM Lin^-^ compartment and impaired CFU-F capability compared to controls, suggesting stem cell exhaustion following prolonged suppression of *Tsc1*. Parallel observations have been reported in HSCs following the somatic deletion of *Tsc1*, where *Tsc1* deficiency was found to promote short-term expansion (1–3 days) followed by long-term depletion (>10 days) of the BM-HSC pool and loss of repopulation potential [9]. In MSCs, direct effects of *Tsc1* on aging have not been reported; however global transcriptional profiling of Lin^-^/CD34^-^/CD31^-^ BM cells isolated from young and elderly women have identified significant mTOR pathway regulation [54]. Similarly, in vitro aging of MSCs has been shown to be attenuated by inhibition of PI3K/Akt/mTOR signaling, resulting in maintenance of clonogenic capacity and high proliferation rates after long-term culture expansion [55].

*Tsc1* inactivation in cultured MSCs also led to ROS accumulation and increased senescence, consistent with premature cell aging [56]. In HSCs, loss of function following *Tsc1* inactivation has been attributed to ROS accumulation and onset of senescence and can be reversed by rapamycin treatment or antagonizing ROS [8,57]. We speculate that prolonged hyperactivation of mTOR similarly drives premature aging of MSCs following *Tagln*-mediated *Tsc1* inactivation. In keeping with this hypothesis, immunostaining of kidney cysts, which were found to exhibit tubular proliferation following *Tsc1* loss, revealed increased levels of markers associated with cellular senescence (p16, p53, and p21), suggesting a concomitant upregulation of anti-proliferative mechanisms. Intriguingly, while the loss of 1 or both alleles of *Tsc1* increased SA β-gal levels, significant p16 staining was observed only in heterozygous mice, suggesting that upregulation of this tumor suppressor was inhibited in response to homozygous *Tsc1* deletion. Interestingly, *Tsc1* deletion was not sufficient to induce transformation, as neither *Tsc1^∆/∆^* (28 day old) nor *Tsc1^∆/+^* (1.5 yr old) mice developed malignant tumors at the time of sacrifice, consistent with the predominantly non-cancerous neoplastic growth characteristic of human TSC. This may be due to the upregulation of p53 following either heterozygous or homozygous *Tsc1* deletion, which can sensitize cells to cellular stress in response to mTOR activation [58].

## 5. Conclusions

**Figure 7:**
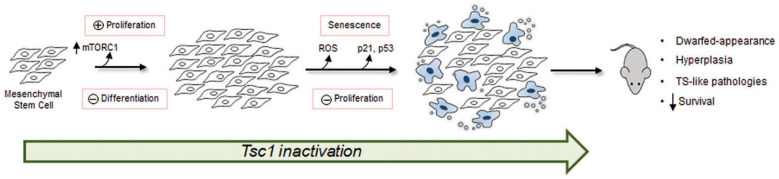
Proposed model showing downstream effects of *Tsc1* inactivation in mesenchymal progenitors. *Tsc1* loss causes transient amplification of the mesenchymal stem cells via hyperactivation of mTORC1. Proliferating cells maintain a progenitor (stem-like) state with impaired differentiation along mesenchymal lineages. Sustained hyperproliferation, during aging or following in vitro expansion, induces ROS accumulation and replicative senescence via the upregulation of p21 and p53. In vivo, *Tsc1* loss culminates in defective skeletal development and hyperplasia affecting multiple organ systems including the heart and kidneys, ultimately reducing lifespan.

Our results show that loss of *Tsc1* function, through targeted genetic deletion or RNA interference induces an mTOR-dependent hyperproliferative phenotype in post-natal BM-MSCs. In aged mice, this phenotype is lost, with reduced colony-forming potential of MSCs following *Tsc1* suppression. *Tsc1* loss also led to impaired differentiation along adipocyte and SM lineages, and the onset of a senescent phenotype in vitro and in vivo. In addition to these findings, *Tagln*-mediated *Tsc1* inactivation generated a phenotype similar to TSC with impaired development of several organs, polycystic kidney disease, and cardiac hypertrophy. Based on these observations, we speculate that maintenance of MSCs in the BM is under the regulatory control of Tsc1-mTOR and that loss of *Tsc1* function causes transient amplification of the progenitor pool followed by premature aging/senescence, and impaired multilineage development (Figure 7). Given the widespread therapeutic potential of MSCs, modulation of TSC1-mTOR provides possible interventions for enhancing their regenerative functions. This may include treatments that expand cell numbers to meet the growing demand for large quantities of MSCs in a variety of clinical applications or to help direct differentiation towards specific cell types for the regeneration of diseased tissues.

## Figures and Tables

**Table 1:** Mouse primer sequences for RT-qPCR.

Gene	Primer Sequences
*Tsc1*	F: 5′-ATGGCCCAGTTAGCCAACATT-3′
	R: 5′-CAGAATTGAGGGACTCCTTGAAG-3′
*Gapdh*	F: 5′-CCTGGAGAAACCTGCCAAGTATG-3′
	R: 5′-AGAGTGGGAGTTGCTGTTGAAGTC-3′
*18S rRNA*	F: 5′-TTGTACACACCGCCCGTCGC-3′
	R: 5′-CTTCTCAGCGCTCCGCCAGG-3′
*Asma*	F: 5′-GAGAAGCCCAGCCAGTCG-3′
	R: 5′-CTCTTGCTCTGGGCTTCA-3′
*Tagln (SM22α)*	F: 5′-TAATGGCTTTGGGCAGTTTG-3′
	R: 5′-TGCAGTTGGCTGTCTGTGAA -3′
*Myh11 (SM-Mhc)*	F: 5′-GCAGAAGGCTCAGACCAAAG-3′
	R: 5′-TATCCAGAATGCCCAGGAAG-3′
*Cebpα*	F: 5′-GCCGAGATAAAGCCAAACAAC-3′
	R: 5′-GACCCGAAACCATCCTCTG-3′
*Cebpβ*	F: 5′-GCCAAGAAGACGGTGGACA-3′
	F: 5′-ACAAGTTCCGCAGGGTGCT-3′
*Pparγ*	F: 5′-TTGCTGAACGTGAAGCCCATCGAGG-3′
	R: 5′-GTCCTTGTAGATCTCCTGGAGCAG-3′

## Supplementary Materials

The following are available online at https://www.mdpi.com/2073-4409/9/9/2072/s1, Figure S1: Specificity of *Tagln*-mediated recombination in LacZ reporter mice; Figure S2. *Tsc1* is inactivated in BM-MSCs following Tagln-mediated recombination.
