# Supplementary material for: Tsc1 Regulates the Proliferation Capacity of Bone-Marrow Derived Mesenchymal Stem Cells
Source: Cells. 2020 Sep 10;9(9):2072. doi: 10.3390/cells9092072 (PMC7565438; doi:10.3390/cells9092072)
Supplement: Supplementary file 1 [file cells-09-02072-s001.pdf]

cells

MDPI

Supplementary figure S1

to manuscript "Tsc1 regulates the proliferation capacity of bone-marrow derived mesenchymal stem cells" by Maria V. Guijarro, Laura S. Danielson, Marta Cañamero, Akbar Nawab, Carolina Abrahan, Eva Hernando and Glyn D. Palmer.

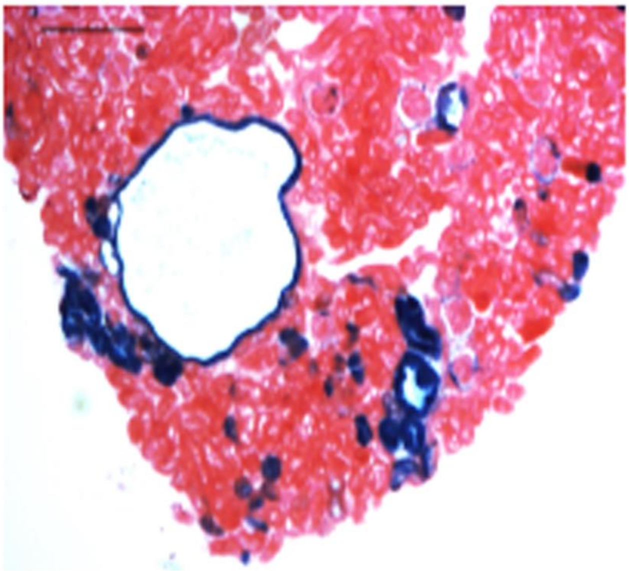

Figure S1. Specificity of *Tagln*-mediated recombination in LacZ reporter mice. Representative image of β-gal staining of a kidney recovered from 28 day old *Tagln*-Cre-lacZ mice to determine tissue specificity of *Tagln*-mediated recombination. β-galactosidase staining was performed on whole mount kidneys following fixation and sections were counterstained with eosin. Positively stained areas were confined to smooth muscle-lined vessel structures and lining cells of cystic lesions. Scale Bar: 250 µm.

Supplementary figure S2

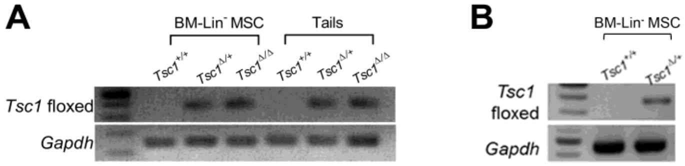

Figure S2. *Tsc1* is inactivated in BM-MSCs following Tagln-mediated recombination. BM lin$^-$ cells were harvested from control and *Tsc1* mutant mice and plated to generate adherent MSC cultures of each genotype. Recombination of *Tsc1* alleles was confirmed by PCR of extracted genomic DNA using primers specific for *Tsc1* floxed loci. **(A)** MSCs from 28 day old mice. Tail biopsy tissue was used as a positive control. **(B)** MSCs from a separate aged, 1.5 yr old cohort. Note that only heterozygous mice $Tsc1^{\Delta/+}$ were used for aging studies as total loss of *Tsc1* ($Tsc1^{\Delta/\Delta}$) induced premature lethality.
